# Aggresome–Autophagy Associated Gene HDAC6 Is a Potential Biomarker in Pan-Cancer, Especially in Colon Adenocarcinoma

**DOI:** 10.3389/fonc.2021.718589

**Published:** 2021-08-17

**Authors:** Zhiyong Zhang, Xin Zhang, Aimin Huang

**Affiliations:** ^1^Department of Colorectal Surgery, The First Affiliated Hospital of Zhengzhou University, Zhengzhou, China; ^2^Department of General Surgery, Medical College of Zhengzhou University, Zhengzhou, China

**Keywords:** pan-cancer, histone deacetylase 6, immune microenvironment, DNA methylation, immunotherapy

## Abstract

**Background:**

Histone deacetylase 6 (HDAC6) regulates cytoplasmic signaling networks through the deacetylation of various cytoplasmic substrates. Recent studies have identified the role of HDAC6 in tumor development and immune metabolism, but its specific function remains unclear.

**Methods:**

The current study determined the role of HDAC6 in tumor metabolism and tumor immunity through a multi-database pan-cancer analysis. The Cancer Genome Atlas (TCGA), Genotype-Tissue Expression (GTEx), and Cancer Cell Line Encyclopedia (CCLE) datasets were used to determine the expression levels, prognosis, tumor progression, immune checkpoints, and immune metabolism of HDAC6 in 33 tumors. Pathways, immune checkpoints, immune neoantigens, immune microenvironment, tumor mutational burden (TMB), microsatellite instability (MSI), DNA mismatch repair (MMR), and the value of methyltransferases. The R package was used for quantitative analysis and panoramic description.

**Results:**

In the present study, we determined that HDAC6 is differentially expressed in pan carcinomas, and by survival, we found that HDAC6 was generally associated with the prognosis of pancreatic adenocarcinoma, Thymoma, and uveal melanoma, where low expression of HDAC6 had a significantly worse prognosis. Secondly, through this experiment, we confirmed that HDAC6 expression level was associated with tumor immune infiltration and tumor microenvironment, especially in PAAD. Finally, HDAC6 was associated with immune neoantigen and immune checkpoint gene expression profiles in all cancers in addition to TMB and MSI in pan-cancers.

**Conclusion:**

HDAC6 is differentially expressed in pan-cancers and plays an essential role in tumor metabolism and immunity. HDAC6 holds promise as a tumor potential prognostic marker, especially in colon cancer.

## Introduction

Living standards, dietary habits, and living environments of people is changing (1), and the incidence of various diseases is exploding (2). Globally, cancer has become a severe public health hazard; cancer incidence and mortality rates are increasing rapidly every year (3). Malignancy is one of the leading causes of death worldwide, and treatment success is low in developed and developing countries (4). Although breakthroughs in tumor diagnosis and tumor treatment have been made in recent years, conventional therapies, including surgery, chemotherapy, and radiation therapy, remain the first-line treatment for most cancer patients (5). However, patients with many cancers still do not achieve the desired level of 5-year survival with treatment (6). Currently, the application of tumor biomarkers has attracted much attention from scholars, among which pan-cancer analysis has emerged as a potential option to explore new tumor biomarkers (7).

Overexpression of histone deacetylases (HDACs) in cancer cells is an important cause of acetylation imbalance. A total of 18 HDACs have been identified in Ijsselsteijn mans, which can be classified into four categories according to their class namely, I (HDAC1, 2, 3, and 8), II (HDAC4, 5, 6, 7, 9, and 10), III (SIRT1, 2, 3, 4, 5, and 6) and IV (HDAC11), of which classes II and III play an essential role in the life course. (HDAC6) is the most widely studied class II histone deacetylase isoform (8), and studies have confirmed that HDAC6 plays an essential role in the liver (9), kidney (10), brain (11), and other areas of the brain (11); in pancreatic (12) tumors the expression of HDAC6 is upregulated. SIRTs, as class III histone deacetylases, play essential roles and broad cellular functions in aging. Recently, it has been shown that HDAC6 and SIRT2 act as deacetylases that regulate the acetylation status of KRAS in cancer cells. SIRT2 also promotes the differentiation and proliferation of intestinal epithelial cells by regulating Wnt-*β*-catenin signaling. Although HDAC6 plays a role in deacetylating histones, recent reports have found that HDAC6 can be involved in tumorigenesis and development through multiple pathways.

Cellular autophagy has emerged as a hot topic in recent years. It has been confirmed that autophagy is associated with tumor metabolic patterns and the formation of tumor heterogeneity. According to its properties, it can be divided into two aspects. On the one hand, autophagy antagonizes the inflammatory response and can inhibit the degree of infiltration of chronic inflammation, thus improving inflammatory cancer transformation. On the other hand, autophagy can act as an essential pathway of cellular energy metabolism and material recycling by degrading damaged mitochondria and assisting tumors in escaping from Reactive Oxygen Species (ROS) during aerobic glycolysis, thus ensuring the sustainability of the Warburg effect. The aggresome–autophagy pathway is a specific type of induced autophagy. In other words, misfolded and aggregated proteins are selectively recognized for reverse transportation to the center of the microtubule tissue, where they form aggresomes in the pericentriolar region and are subsequently cleared by autophagy. An increasing number of studies have identified the aggresome–autophagy pathway as a critical cellular defense system to prevent the accumulation of misfolded and aggregated proteins. Recent studies have found that HDAC6 is closely associated with aggresome–autophagy and that cells lacking HDAC6 cannot form an aggresome and are highly sensitive to the accumulation of misfolded proteins. It may be a potential mechanism for the involvement of HDAC6 in autophagy.

In addition, HDAC6 activity can also affect the gene expression of several critical immune molecules. These include programmed death receptor-1 (PD-1) and programmed death receptor ligand-1 (PD-L1), tumor-associated antigens, and these factors are central targets for cancer immunotherapy (13).

Unfortunately, the mechanism of HDAC6 in tumor metabolism and tumor immunity is not yet precise. In the present study, we used multiple datasets, such as Cancer Cell Lineage Encyclopedia (CCLE) and The Cancer Genome Atlas (TCGA) for pan-cancer analysis to reveal the precise mechanism of HDAC6. With the rise of high-throughput sequencing, histology technologies are gradually coming into the limelight. New perspectives for cancer research have been provided through histological technologies.

In this study, we analyzed HDAC6 expression, prognosis, TMB, and MSI in 33 tumors by pan-cancer analysis. In addition, we also examined the correlation of HDAC6 in an immune microenvironment, immune-related antigens, and checkpoint genes. We further confirmed that HDAC6 expression affected the expression of DNA repair genes and methyltransferases in pan-cancer. By gene set enrichment analysis, we found that HDAC6 regulated signaling pathways related to cancer apoptosis and tumor immunity.

## Methods and Materials

### Sample Source

Studies were based on The Cancer Genome Atlas (TCGA, https://portal.gdc.cancer.gov/) dataset (14), Genotype-Tissue Expression (GTEx, https://gtexportal.org/) dataset (15), Cancer Cell Line Encyclopedia (CCLE, https://portals.broadinstitute.org/) dataset (16) and the timer (https://cistrome.shinyapps.io/timer/) dataset (17). The TCGA (**Table 1**) and GTEx datasets were used to obtain gene expression information of tumor and normal samples and clinical information data and to analyze HDAC6 expression in 27 tumors. HDAC6 expression in 31 cancers in the GTEx dataset was also analyzed. To examine differential gene expression in cancers on a larger scale, RNA sequencing datasets for each cell line in the CCLE dataset were downloaded, while cancer immune infiltrating cell score data were downloaded through the timer dataset for tumor immune infiltration analysis. In this study, only open access data were used, which precluded the requirement for ethics committee approval.

**Table 1:** TCGA Study Abbreviations in manuscript.

Tumor Name	Abbreviations
Adrenocortical carcinoma	ACC
Bladder urothelial carcinoma	BLCA
Breast invasive carcinoma	BRCA
Cervical squamous cell carcinoma and endocervical adenocarcinoma	CESC
Cholangiocarcinoma	CHOL
Colon adenocarcinoma	COAD
Rectum adenocarcinoma	READ
Lymphoid neoplasm diffuse large B-cell lymphoma	DLBC
Esophageal carcinoma	ESCA
Glioblastoma multiforme	GBM
Head and neck squamous cell carcinoma	HNSC
Kidney chromophobe	KICH
Kidney renal clear cell carcinoma	KIRC
Kidney renal papillary cell carcinoma	KIRP
Acute myeloid leukemia	LAML
Low grade glioma	LGG
Liver hepatocellular carcinoma	LIHC
Lung adenocarcinoma	LUAD
Lung squamous cell carcinoma	LUSC
Mesothelioma	MESO
Ovarian serous cystadenocarcinoma	OV
Pancreatic adenocarcinoma	PAAD
Pheochromocytoma and paraganglioma	PCPG
Prostate adenocarcinoma	PRAD
Rectum adenocarcinoma	READ
Sarcoma	SARC
Skin cutaneous melanoma	SKCM
Stomach adenocarcinoma	STAD
Stomach and esophageal carcinoma	STES
Testicular germ cell tumors	TGCT
Thyroid carcinoma	THCA
Thymoma	THYM
Uterine corpus endometrial carcinoma	UCEC
Uveal melanoma	UVM

### Analysis of HDAC6 Expression Levels and Prognosis in Pan-Cancer

In this study, the R package (edgeR) was used to analyze the differential levels of HDAC6 expression in the dataset. For the expression of HDAC6 in different tumor cells and other normal tissues, we used Kruskal–Wallis test line analysis, and the R package ggplot presented the figures. For the predictive analysis of HDAC6 in pan-cancer, a one-way Cox regression test was used to analyze the correlation between HDAC6 and patient survival. The Kaplan–Meier (K–M) test was also used to compare the survival of patients. The images were plotted using forest plot visualization software.

### Gene Set Enrichment Analysis

Gene set enrichment analysis (GSEA) is a powerful analysis method for interpreting gene expression data and analyzing statistically significant and consistent differences between different groups with different biological states (18). The signaling pathway of HDAC6 was analyzed by gene set enrichment analysis. The Kyoto Encyclopedia of Genes and Genomes (KEGG) pathway enrichment analysis was performed with the R package clusterProfiler. Another dataset used for GSEA analysis is the Molecular Signature Database (MsigDB) (19), in which the Hallmark gene set was used. Implementation criteria were | NES | >1, p-value <0.05, FDR <0.25.

### Immune Checkpoint Genes and Immune Neoantigen Analysis

Immune checkpoints are a set of molecules expressed on immune cells that regulate the degree of immune activation by maintaining their normal immune function *in vivo* (20). We analyzed the relationship between HDAC6 expression levels and the expression of 47 common immune checkpoint genes. Correlations between HDAC6 expression levels and expression of various immune checkpoint genes in multiple cancers were assessed by the R package (limma, reshape2, RColorBrewer). The number of neoantigens in each tumor sample was counted and detected using a scanner.

### Correlation Analysis of HDAC6 in Immune Infiltration and Tumor Microenvironment

To evaluate the performance of HDAC6 gene in immune infiltration, we performed correlation analysis between HDAC6 and six immune cell infiltrations with the help of purity-adjusted Spearman. In addition, we performed an ESTIMATE algorithm by standardized expression matrix to assess the tumor microenvironment-related scores of patients, including estimation score, stromal score, and immune score. p-value <0.05 was considered statistically significant.

### Tumor Mutational Burden and Microsatellite Instability Analysis

The Perl language and R package (fmsb) were used to calculate the total TMB and analyze its correlation with HDAC6 expression levels in patients with pan-cancer. MSI has novel microsatellite alleles in tumors compared to normal tissue and can be used for screening, diagnosis, and prognosis of pancreatic cancer (21). The correlation between MSI and HDAC6 expression levels in pan-cancer was analyzed using the R language with the fmsb package.

### DNA Mismatch Repair Gene Mutation and DNA Methylation Analysis

The correlation between MMR gene expression levels and HDAC6 expression levels was analyzed using the Spearman correlation method. In addition, DNA methylation is an essential factor affecting gene expression. In this study, we evaluated the expression levels of DNMT1, DNMT2, DNMT3A, and DNMT3B and assessed the correlation between these four methyltransferases and HDAC6 expression using the Spearman correlation method.

## Results

### HDAC6 Expression Was Significantly Upregulated in Pan-Cancer

**Figure 1:**
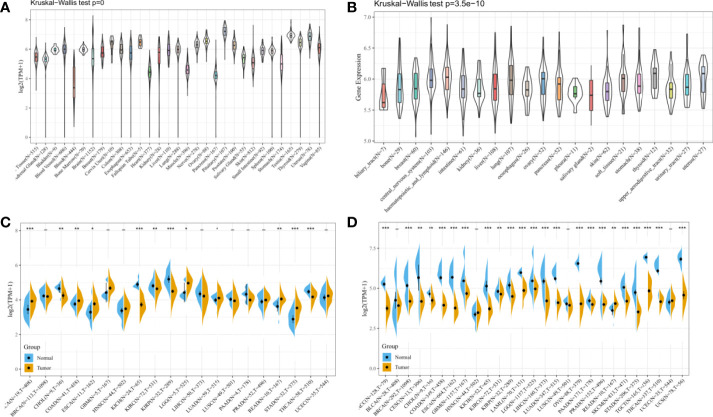
Expression levels of HDAC6 in pan-cancer. **(A)** Analysis of HDAC6 expression levels in pan-cancer based on the GTEx dataset. **(B)** Analysis of HDAC67nbsp;expression levels in various cancer cells based on the CCLE dataset. **(C)** Analysis of HDAC6 expression levels in pan-cancer based on TCGA database. **(D)** Integration of GTEx and TCGA databases to obtain 27 tumor modules and to analyze HDAC6 expression levels in tumor modules. * indicates P < 0.05 compared with control, ** indicates P < 0.01 compared with control, *** indicates P < 0.001 compared with control.

In this study, to observe the expression level of HDAC6 in pan-cancer, the expression of HDAC6 in various cancer databases was first analyzed. The expression levels of HDAC6 in the GTEx (**Figure 1A**), CCLE (**Figure 1B**), and TCGA (**Figure 1C**) datasets were shown by a histogram. Considering the small number of normal sample data in TCGA, the expression of HDAC6 in each cancer in the TCGA and GTEx datasets was further integrated. By integration, 27 tumor modules were retained, and the final analysis showed that HDAC6 expression was downregulated in the combined 27 tumor modules (**Figure 1D**).

### Prognostic Analysis of HDAC6 in Pan-Cancer

**Figure 2:**
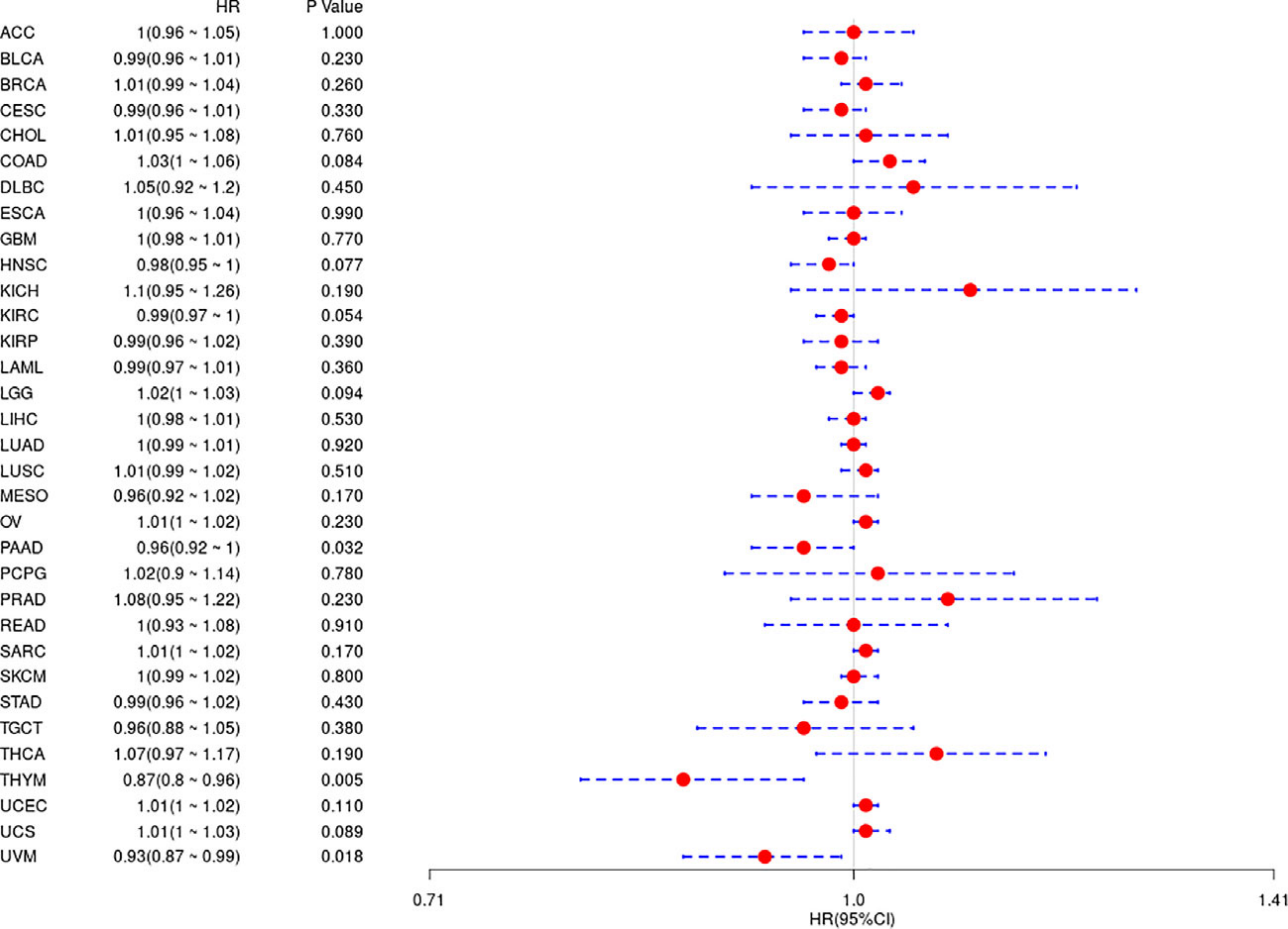
Analysis of the relationship between HDAC6 and OS in 33 tumors using one-way Cox regression, presented using forest plots.

**Figure 3:**
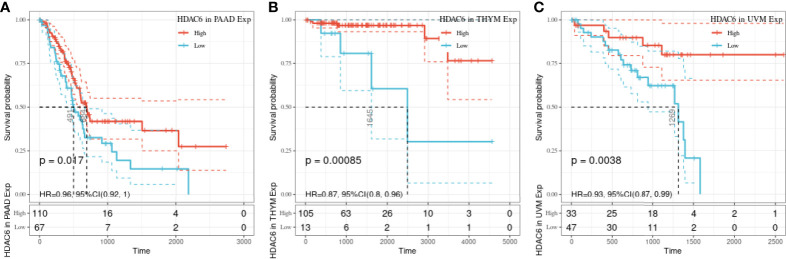
Relationship between HDAC6 and PAAD, THYM, and UVM OS. **(A)** The relationship between HDAC6 high and low expression levels and PAAD OS was analyzed using K–M survival. **(B)** Relationship between HDAC6 high and low expression levels and THYM OS using K–M survival analysis. **(C)** The relationship between HDAC6 high and low expression levels and UVM OS using K–M survival analysis.

**Figure 4:**
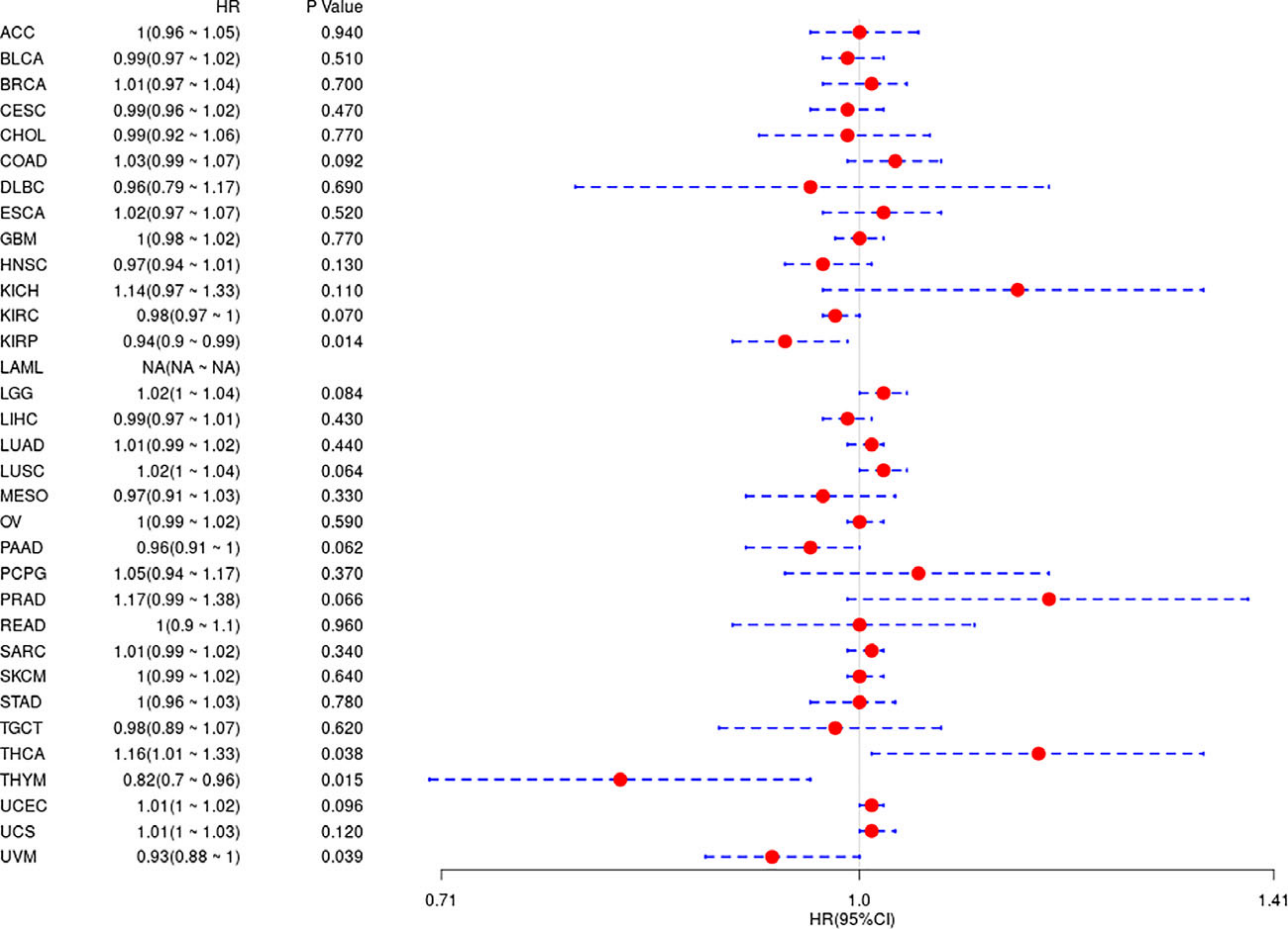
Analysis of the relationship between HDAC6 and DSS in 33 tumors using one-way Cox regression, presented using forest plots.

**Figure 5:**
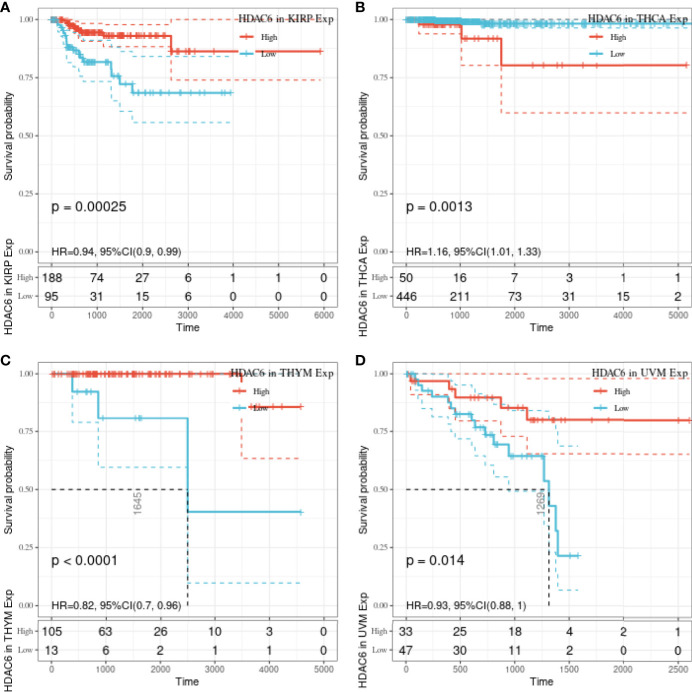
Relationship between HDAC6 and KIRP, THCA, THYM, UVM DSS. **(A)** The relationship between HDAC6 high and low expression levels and KIRP DSS was analyzed by K–M survival. **(B)** The relationship between HDAC6 high and low expression levels and THCA DSS was analyzed using K–M survival. **(C)** Relationship between HDAC6 high and low expression levels and THYM DSS using K–M survival analysis. **(D)** Relationship between HDAC6 high and low expression levels and UVM DSS using K–M survival analysis.

In the above study, the expression levels of HDAC6 in various tumor tissues were determined. To understand the relationship between HDAC6 and tumor prognosis, one-way Cox regression analysis was used to analyze HDAC6 expression levels and patient prognosis. Based on the TCGA database data, HDAC6 expressions were grouped into high and low expression groups according to the median value of HDAC6 expression in each tumor. It was further observed that HDAC6 was only expressed in 33 tumors in PAAD (HR = 0.960, 95CI%: 0.990–1.000, P = 0.032), THYM (HR = 0.870, 95CI%: 0.800–0.960, P = 0.005), and UVM (HR = 0.930, 95CI%: 0.870–0.990, P = 0.018) with of prognostic significance (**Figure 2**). In addition, the relationship between HDAC6 and PAAD, THYM, and UVM was further observed by plotting K–M survival curves (**Figure 3**). It suggested that there was an association between HDAC6 and tumor prognosis, especially with THYM. Considering the possible existence of non-tumor-related deaths during follow-up, we analyzed the relationship between HDAC6 expression levels and prognostic DSS (disease-specific survival) in 33 tumors of TCGA. The results of the analysis showed that HDAC6 was associated with KIRP (HR = 0.940, 95CI%: 0.900–0.990, P = 0.014), THCA (HR = 1.16, 95CI%: 1.010–1.330, P = 0.038), THYM (HR = 0.820, 95CI%: 0.700–0.960, P = 0.015), and UVM (HR = 0.870, 95CI%: 0.800–0.960, P = 0.005), indicating that HDAC6 and DSS had prognostic significance (**Figure 4**). Also, the K–M survival curve indicated that HDAC6 was a prognostic indicator of tumor DSS (**Figure 5**).

### Tumor Metabolic and Immune Signaling Pathways Involved in HDAC6

**Figure 6:**
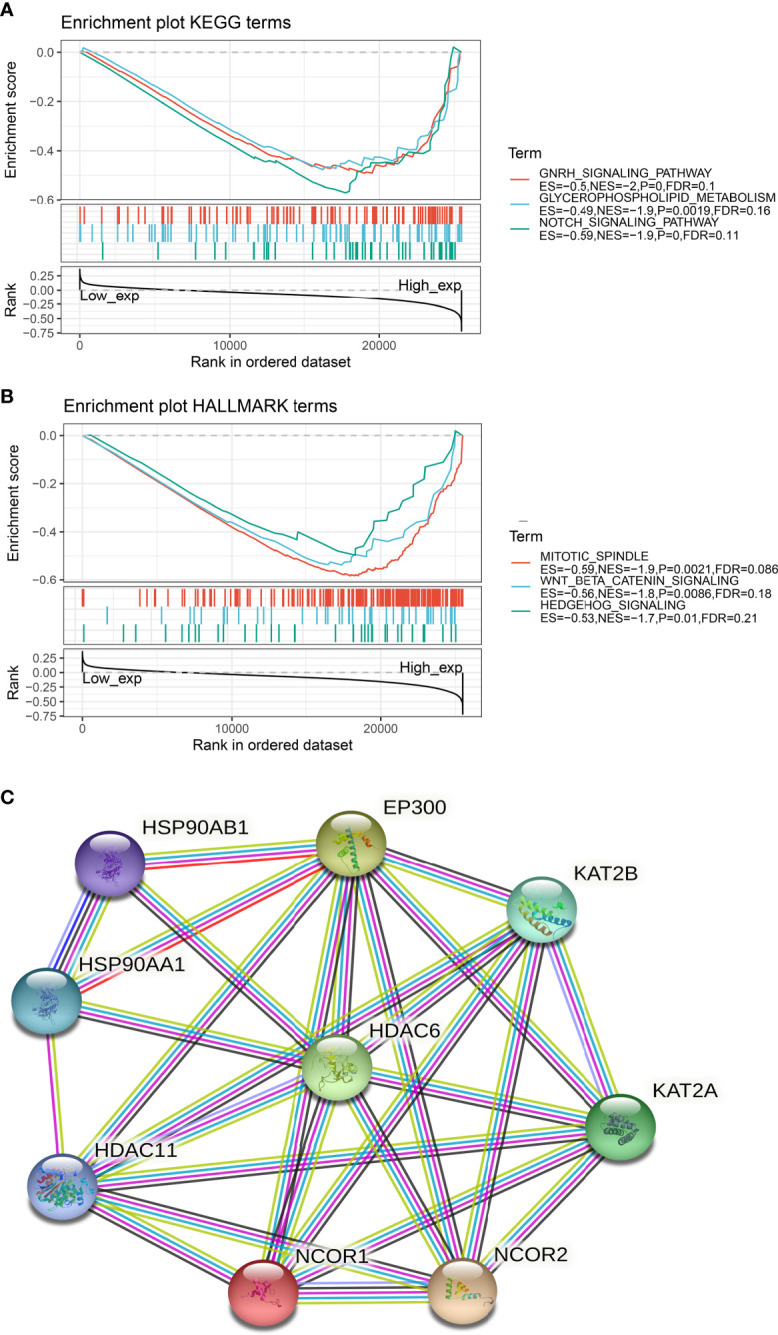
Signaling enrichment of HDAC6 in KEGG and markers. **(A)** GSEA analysis of the top three correlations of HDAC6 with signaling pathways in the KEGG database. **(B)** GSEA analysis of HDAC6 with the top three results of signaling pathway correlations in the marker dataset. **(C)** String constructs HDAC6 protein co-expression network.

To further understand the relationship between HDAC6 involvement in cancer metabolism and tumor immunity, a GSEA analysis was performed to analyze the signaling enrichment of KEGG and markers in both groups according to HDAC6 gene expression, which was divided into high and low groups. The results were ranked according to NES scores for the top ten most enriched signaling pathways or biological processes (**Tables S1**, **S2**). In addition, we mapped the top three most abundant signaling pathways in both databases. Among them, we found that the Notch signaling pathway, Wnt/*β*-catenin signaling pathway, and Hedgehog signaling pathway were the most enriched (**Figures 6A, B** **)**.

In addition, we constructed an HDAC6 PPI (protein–protein interaction) network and found that HDAC6 was related to eight proteins (**Figure 6C**).

### Correlation Between HDAC6 Expression Levels and Immune Checkpoints and Immune Neoantigens in Pan-Cancer

**Figure 7:**
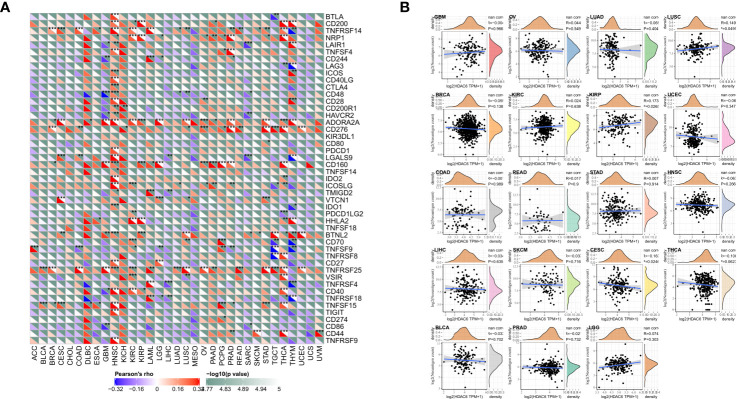
Correlation analysis of HDAC6 expression levels with immune checkpoints and immune neoantigens. **(A)** Correlation analysis of HDAC6 expression levels with immune checkpoint gene expression levels in various tumors. **(B)** Correlation analysis of HDAC6 expression levels in various tumors with the number of neoantigens in tumors. * indicates correlation difference of P < 0.05, ** indicates correlation difference of P < 0.01, *** indicates correlation difference of P < 0.001.

Immune checkpoints are a series of molecules expressed in immune cells that regulate the degree of immune activation and play an essential role in regulating autoimmunity (22). In contrast, tumor neoantigens are nascent antigens encoded by mutated genes in tumor cells. The synthesis of neoantigen vaccines is facilitated by exploiting the immune activity of tumor neoantigens (23). This time, to explore the relationship between HDAC6 and immune regulation, it was preferred to analyze the correlation between HDAC6 and immune checkpoints and immune neoantigens. Our results found that HDAC6 expression levels in various tumors correlated with more than 40 checkpoints, with a positive correlation mainly with HNSC, THCA (**Figure 7A**). It suggested that HDAC6 had a role in modulating these immune checkpoints in some tumors and may have the ability to strengthen immunity. In addition, in the present study, we calculated the number of neoantigens for each tumor type. The results showed that HDAC6 was only correlated in KIPR (R = 0.173, P = 0.027), and CESE (R = −0.163, P = 0.025) (**Figure 7B**).

### Correlation Between HDAC6 and Tumor Immune Infiltration and the Tumor Microenvironment in Pan-Cancer

**Figure 8:**
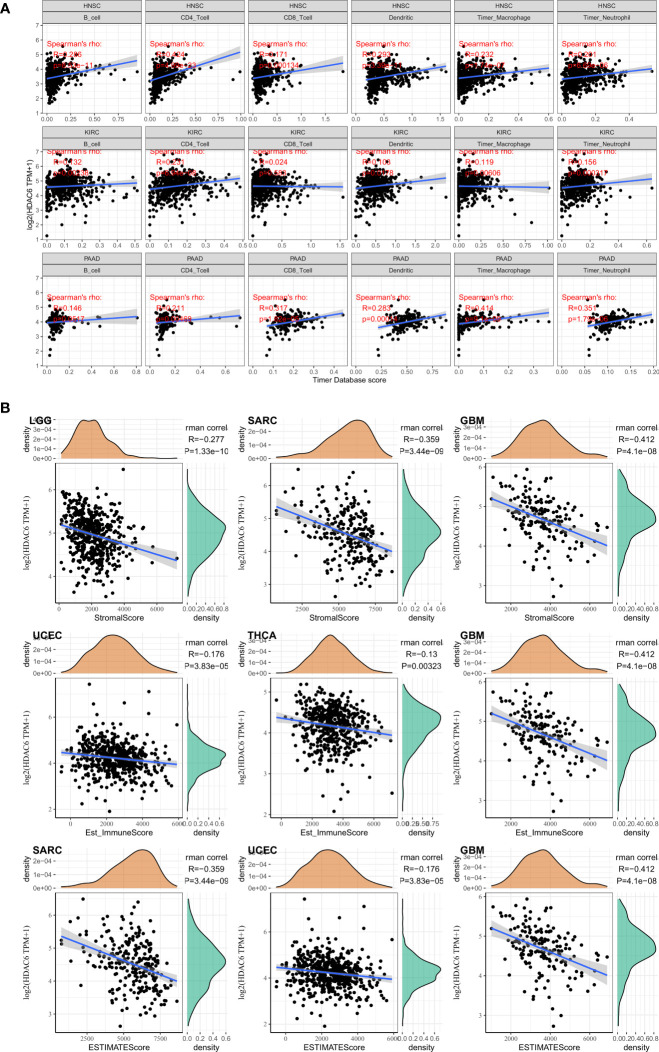
correlation between HDAC6 and tumor immune infiltration and tumor microenvironment. **(A)** Correlation analysis between HDAC6 and immune cell infiltration in HNSC, KIRC, PAAD. **(B)** Relationship between HDAC6 expression levels and immune scores, stromal scores, and immune scores of ESTIMATE.

Differences in the degree of infiltration of different immune cells are highly correlated with tumor progression and prognosis (24). In contrast, the tumor microenvironment consists of various cells, external matrix, and associated factors that significantly influence tumor diagnosis, survival outcome, and clinical treatment sensitivity (25). In the present study, we analyzed immune infiltration and tumor immune microenvironment of HDAC6 in different cancers. A total of 3 most relevant tumors (HNSC, KIRC, PAAD) were identified after analyzing 33 tumors in the TCGA database. Among these tumors, HDAC6 was positively correlated with B cells (R = 0.286, P < 0.001), CD4+ T cells (R = 0.424, P < 0.001), neutrophils (R = 0.293, P < 0.001), macrophages (R = 0.232, P < 0.001), and dendritic cells (R = 0.201, P < 0.001) in HNSC. In contrast, HDAC6 was positively correlated only with CD4+ T cells in KIRC (R = 0.231, P < 0.001). In addition, HDAC6 was positively correlated with CD4+ T cells (R = 0.211, P = 0.005), CD8+ T cells (R = 0.317, P < 0.001), neutrophils (R = 0.283, P < 0.001), macrophages (R = 0.414, P < 0.001) and dendritic cells (R = 0.351, P < 0.001) in PAAD (**Figure 8A**). In addition to analyzing the relationship between HDAC6 and tumor immune infiltration, the present study also analyzed the correlation between HDAC6 and tumor microenvironment in pan-cancer. Individual tumor samples were analyzed by R package (ESTIMATE), and the relationship between HDAC6 expression levels and immune score, stromal score, and the immune score of ESTIMATE was analyzed separately. We showed the top three tumors in which LGG (R = −277, p < 0.001), SARC (R = −0.359, p < 0.001), BGM (R = −0.412, p < 0.001) immune scores were negatively correlated with HDAC6 expression levels in 33 tumors. UCEC (R = −0.176, P < 0.001), THCA (R = −0.13, P = 0.003), BGM (R = −0.412, P < 0.001) stromal scores were negatively correlated with HDAC6 expression levels. In addition, the immune scores of SARC (R = −0.359, P < 0.001), UCEC (R = −0.176, P < 0.001), BGM (R = −0.412, P < 0.001) ESTIMATE were found to be negatively correlated with HDAC6 expression levels (**Figure 8B**). The above results suggested that HDAC6 was negatively correlated with both tumor immune scores.

### TMB and MSI Analyses of HDAC6 Expression Levels in Pan-Cancer

**Figure 9:**
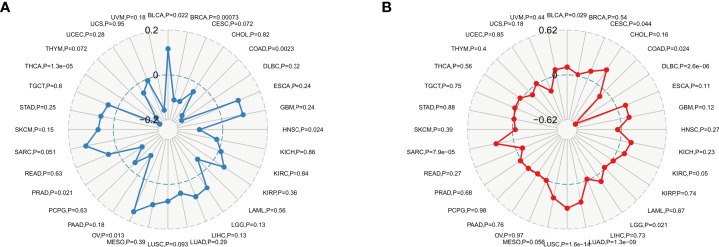
TMB and MSI analysis of HDAC6 expression level *vs*. **(A)** Spearman test to analyze the relationship between HDAC6 and TMB in pan-cancer. **(B)** Spearman test to analyze the relationship between HDAC6 and MSI in pan-cancer.

TMB has a high value in predicting the efficacy and prognosis of immune checkpoint therapy to assess the total number of substitutions and insertion/deletion mutations per megabase in the exon coding regions of genes in tumor samples (26). MSI is caused by defects in mismatch repair (MMR) genes and is strongly associated with tumorigenesis (21). In the present study, we analyzed the relationship between HDAC6 in TMB and MSI. Analysis by Spearman test revealed that HDAC6 expression levels were positively correlated with BLCA, COAD, and OV, and negatively correlated with BRCA, HNSC, PRAD, and THCA, respectively (**Figure 9A**). In addition, Spearman test analysis found HDAC6 expression levels in tumors given in the MSI. HDAC6 expression levels were positively correlated with BLCA, CESC, COAD, LGG, LUAD, LUSC, and SARC, respectively, and negatively correlated with DLBC by Spearman test analysis (**Figure 9B**).

### Relationship Between HDAC6 Expression Level and MMR Gene and Methyltransferase in Pan Carcinoma

**Figure 10:**
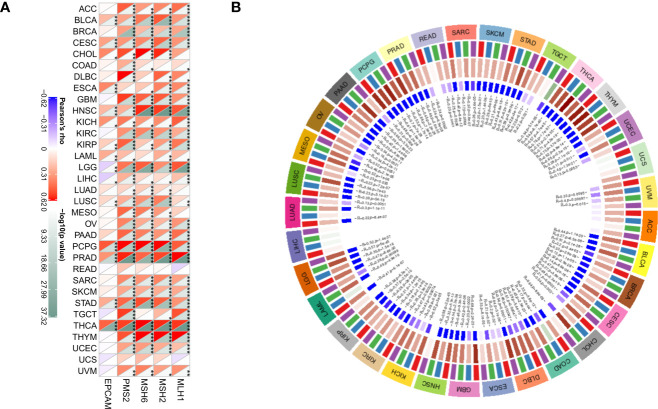
Correlation of HDAC6 expression level with MMR gene and methyltransferase. **(A)** Spearman test to analyze the correlation between HDAC6 expression level and MMR gene in various tumors. **(B)** Spearman test to analyze the correlation between HDAC6 expression level and methyltransferase in various tumors. * Indicates that EPCAM, PMS2, MSH6, MSH2, MLH1 and HDAC6 are correlated in tumors, P<0.05. **Indicating that EPCAM, PMS2, MSH6, MSH2, MLH1 and HDAC6 are correlated in tumors, P< 0.01. *** means EPCAM, PMS2, MSH6, MSH2, MLH1 and HDAC6 are correlated in tumors, P<0.001.

DNA methylation has a role in altering the structure and stability of DNA, and the appearance of MMR leads to mutations in somatic cells that are more deleterious (27). DNA methyltransferases are involved in the occurrence of methylation and play an essential regulatory role in the methylation process (28). In the present study, the relationship between HDAC6 and MMR genes (MLH1, MSH2, MSH6, PMS2, EPCAM) in various tumors was evaluated according to the TCGA database,. The analysis showed that HDAC6 expression levels were positively correlated with MMR genes in all 33 tumors except READ and UCS (**Figure 10A**). In addition, further investigation revealed that HDAC6 expression levels were significantly and positively correlated with both methyltransferase expression levels (**Figure 10B**). These results suggest that HDAC6 is involved in tumorigenesis development and has epigenetic properties that regulate various tumors.

## Discussion

The number of cancer cases worldwide has exceeded 90 million, and cancer is currently a more prominent public health problem (3). A pan-cancer analysis mines genomic similarities and differences in tumors through major databases to provide better insights about cancer diagnosis and treatment to guide cancer-related research (29).

HDAC6 is a unique member of the HDAC family located on chromosome Xp11.23 and is the giant protein molecule in the HDAC family (30). The primary role of HDAC6 is to acetylate and deacetylate histones, which are modified by histone acetyltransferase (HAT) and histone deacetylase (HDAC). HAT has a role in promoting chromosome depolymerization and activating transcription, while HDAC has a role in blocking DNA and inhibiting transcription. Loss-of-nest apoptosis is a specific type of programmed cell death that results from the detachment of cells from the extracellular matrix and surrounding basement membrane. Recent studies have found that HDAC6 promotes oncogenic transformation and tumor formation by promoting anchorage-independent proliferation in transduced cells. In addition to histone regulation, HDAC6 also affects tumor cell motility by regulating non-histone substrates. *α*-microtubulin was the first non-histone substrate identified for HDAC6, and the reversible deacetylation of *α*-microtubulin by HDAC6 affects microtubulin. The reversible deacetylation of *α*-microtubulin by HDAC6 affects microtubule stability and function. It has been found that HDAC6 is highly expressed in melanoma, and by knocking down HDAC6 acetylated *α*-microtubulin increases, acetylated microtubules accumulate, causing CYLD to get around the nucleus, ultimately leading to a reduction in the interaction between CYLD and BCL3, preventing the transcriptional activity of the nuclear factor NF-*κ*B, which in turn affects cell growth and metastasis.

Immunomodulation is of interest as a new idea in cancer therapy, especially in tumors lacking specific molecular targets. TME comprises pro and anti-cancer immune cells containing CD8-effective T cells, natural killer (NK) cells, macrophages, regulatory T cells, and myeloid-derived suppressor cells. Studies have shown a strong link between HDAC6 and the immune microenvironment. For example, inhibition of HDAC6 enhances antitumor immune signaling and reduces tumor load in ovarian cancer (31). HDAC6 has a regulatory antitumor immune response in breast cancer and plays an atypical role in disseminated and invasive breast cancer (32). Although anti-programmed death-1 (PD-1)/programmed death ligand-1 (PD-L1) drugs have achieved considerable clinical efficacy and low toxicity, they are not effective in all cancer types or do not achieve the desired effect in all patients (33). In a study, a novel HDAC6 inhibitor (MPT0G612), which induces apoptosis and inhibits IFN-*γ*-induced programmed death-ligand 1 in human colorectal cancer cells, was found to be a potential strategy for the combination of immune checkpoint inhibitors in the treatment of CRC (34). It suggests that HDAC6 holds promise as a new drug target for anti-cancer immunotherapy or in combination with known immune checkpoint inhibitors to enhance immune infiltration and response to cancer. Therefore, we analyzed HDAC6 expression in pan-cancer, prognosis, immune microenvironment, immune-associated antigens, and checkpoint genes for correlation analysis.

In this study, we first analyzed the expression and prognosis of HDAC6 in pan-cancer. After analyzing GTEx, CCLE, and TCGA databases, we found that HDAC6 was differentially expressed in the TCGA database for pan-cancers. However, considering the small number of control samples in the TCGA database, we integrated samples from the TCGA and GTEx databases. Here the results were reversed, and the expression of HDAC6 in the integrated database for pan-cancers was low, contrary to recent findings (31, 35). We speculate that this may be related to the recent addition of sample data to the TCGA database. Also in our prognostic analysis, we found that patients with low HDAC6 expression in PAAD, THYM, and UVM had significantly lower OS according to HDAC6 expression level which was further divided into high and low expression groups, and patients with low HDAC6 expression in KIRP, THYM, and UVM had significantly lower DSS. These results indicate that HDAC6 is expected to be a prognostic indicator in tumors as mentioned earlier, but further clinical validation is needed.

Cancer metabolism and tumor immune signaling pathways are important areas in basic tumor research (34, 36). To better observe the mechanism of HDAC6 in tumors, the analysis by GSEA revealed that HDAC6 is associated with the Notch signaling pathway (37), Wnt/*β*-catenin signaling pathway (38), and Hedgehog signaling pathway (39) were the most enriched. As the three most relevant pathways to tumor development, single-gene enrichment allowed identifying HDAC6 as involved in the feedback of these pathways and involved in tumorigenesis based on these pathways. In addition, by constructing a PPI protein co-expression network, eight genes were co-expressed with HDAC6, among which HDAC11 was of interest. HDAC11 belongs to class IV histone deacetylation, and earlier studies found that HDAC6 and HDAC11 act as a common transcriptional activator to regulate IL-10 expression, suggesting a possible regulatory role of HDAC6 in cellular immunity.

We found HDAC6 expression correlated with more than 40 checkpoints in the follow-up study, which were not lacking essential immune genes such as PD-1, TIGIT, TNFRSF9, and CTLA4. These results strongly suggest that HDAC6 may be a potential biomarker and play a crucial role in tumor immunity. The immune microenvironment is an essential component in tumor development, and tumor growth and metastasis can be altered by regulating the infiltration of immune cells and immune modifications and immune escape in the tumor microenvironment (40). In addition, the present study also found that HDAC6 was involved in the immune infiltration of tumors, and there was a positive correlation between the expression level of HDAC6 and immune score. They suggested that HDAC6 may be a potential immunosuppressant.

TMB and MSI scores have a high value in predicting the efficacy and prognosis of immune checkpoint therapy (41). With the continuous improvement of immune checkpoints in recent years, immunosuppressants are increasingly used in tumor immunotherapy and have played an influential role in clinical practice (42). In addition to this, PD-1/PD-L1 immunosuppressants have become a first- or second-line treatment option for some tumors (43). In our study, HDAC6 expression levels were shown to correlate with TMB and MSI in four cancer types, respectively. Among them, BLCA and COAD were positively correlated in TMB and MSI with overlapping relationships, and COAD was more significantly associated with TMB and MSI. In recent years, studies on COAD and HDAC6 have been increasing, and Yang et al. (12) found that HDAC6 in colon cancer cells regulates the deacetylation of K-RAS genes in the acetylated state and through the RAS/MAPK signaling pathway. KRAS-activating mutations are present in more than 40% of patients with colon cancer. The recent finding that KRAS mutations in LUAD may serve as a potential predictor for guiding anti-PD-1/PD-L1 immunotherapy needs to be further validated in colon cancer.

We concluded the study by analyzing the relationship between HDAC6 expression levels and the MMR gene and methyltransferase. Under normal circumstances, mismatches may occur during DNA replication, leading to genetic mutations, but regulation by the MMR genome in cells can identify and correct mutations (44). Moreover, mutations in the MMR genome can exacerbate the increased accumulation of genetic errors causing genomic instability or MSI (45). DNA methylation is an essential factor leading to altered tumor development, and an increasing number of studies show that hypermethylation of gene promoters is a common epigenetic feature of cancer (46). In the present study, we found that HDAC6 expression was strongly associated with mutation levels in five MMR genes in human pan-cancers, but there were some exceptions in tumors (READ and UCS). These results are consistent with our conclusion that high HDAC6 expression plays an essential regulatory role in tumor development by regulating MMR gene mutations through DNA methylation.

In the present study, we identified the potential value of HDAC6 in pan-cancer by primary analysis. However, limitations of the study still exist. First, as a primary analysis, we did not perform validation analysis on clinical samples or animal models, such as HDAC6 expression in multiple cancers. The results in the current study differed from recent studies. Second, although we determined that HDAC6 expression levels were associated with tumor immune cell infiltration and patient survival, we could not directly demonstrate that HDAC6 affects patient survival through immune infiltration. Finally, the current study used sequencing data from multiple databases and microarray data for analysis, which are subject to systematic bias. Therefore, we hope to collect clinical samples in future studies and conduct primary research to refine our findings.

Despite the flaws of the current study, we have to acknowledge the close association of HDAC6 in tumor immunity and cancer development. In the present study, we determined that HDAC6 was differentially expressed in pan-cancers and that abnormal expression was associated with tumor progression, especially in COAD. Abnormal expression of HDAC6 was associated with immune checkpoints, immune cell infiltration, tumor microenvironment, TMB, MSI, and DNA methylation. HDAC6 holds promise as a tumor potential prognostic marker, especially in colon cancer.

## Data Availability Statement

Publicly available datasets were analyzed in this study. This data can be found here: https://portal.gdc.cancer.gov/, https://gtexportal.org/, https://portals.broadinstitute.org/, https://cistrome.shinyapps.io/timer/.

## Author Contributions

ZZ: conception and design, writing the article, critical revision of the article. XZ: data collection, writing the article, critical revision of the article, analysis and interpretation. AH: conception and design, writing the article, critical revision of the article. All authors contributed to the article and approved the submitted version.

## Conflict of Interest

The authors declare that the research was conducted in the absence of any commercial or financial relationships that could be construed as a potential conflict of interest.

## Publisher’s Note

All claims expressed in this article are solely those of the authors and do not necessarily represent those of their affiliated organizations, or those of the publisher, the editors and the reviewers. Any product that may be evaluated in this article, or claim that may be made by its manufacturer, is not guaranteed or endorsed by the publisher.
