# Supplementary material for: Aggresome–Autophagy Associated Gene HDAC6 Is a Potential Biomarker in Pan-Cancer, Especially in Colon Adenocarcinoma
Source: Front Oncol. 2021 Aug 17;11:718589. doi: 10.3389/fonc.2021.718589 (PMC8416150; doi:10.3389/fonc.2021.718589)
Supplement: Supplementary file 1 [file Table_1.doc]

| Term | Size | ES | NES | NOM P-val | FDR Q-val |
| --- | --- | --- | --- | --- | --- |
| KEGG_GNRH_SIGNALING_PATHWAY | 101 | -0.5013352 | -1.9984499 | 0 | 0.0996532 |
| KEGG_GLYCEROPHOSPHOLIPID_METABOLISM | 77 | -0.4860993 | -1.9258248 | 0.001919386 | 0.16282876 |
| KEGG_NOTCH_SIGNALING_PATHWAY | 47 | -0.586002 | -1.9249557 | 0 | 0.111097895 |
| KEGG_INOSITOL_PHOSPHATE_METABOLISM | 54 | -0.5590302 | -1.9169043 | 0.001996008 | 0.09041146 |
| KEGG_MTOR_SIGNALING_PATHWAY | 52 | -0.5350731 | -1.9166095 | 0 | 0.07253558 |
| KEGG_INSULIN_SIGNALING_PATHWAY | 137 | -0.47181818 | -1.890206 | 0 | 0.080021724 |
| KEGG_GAP_JUNCTION | 89 | -0.48172438 | -1.8259852 | 0.001996008 | 0.12796791 |
| KEGG_PHOSPHATIDYLINOSITOL_SIGNALING_SYSTEM | 76 | -0.49816334 | -1.8045728 | 0 | 0.13668086 |
| KEGG_SPLICEOSOME | 126 | -0.60187435 | -1.8024412 | 0.020703934 | 0.12325358 |
| KEGG_NON_SMALL_CELL_LUNG_CANCER | 54 | -0.51184565 | -1.8021003 | 0.008064516 | 0.111416884 |

Table S1 The top 10 most enriched KEGG signaling pathways in GSEA
