# Supplementary material for: Aggresome–Autophagy Associated Gene HDAC6 Is a Potential Biomarker in Pan-Cancer, Especially in Colon Adenocarcinoma
Source: Front Oncol. 2021 Aug 17;11:718589. doi: 10.3389/fonc.2021.718589 (PMC8416150; doi:10.3389/fonc.2021.718589)
Supplement: Supplementary file 2 [file Table_2.doc]

| Term | Size | ES | NES | NOM P-val | FDR Q-val |
| --- | --- | --- | --- | --- | --- |
| HALLMARK_MITOTIC_SPINDLE | 197 | -0.5867757 | -1.9474298 | 0.002136752 | 0.08579598 |
| HALLMARK_WNT_BETA_CATENIN_SIGNALING | 42 | -0.5556695 | -1.7995158 | 0.00862069 | 0.18479925 |
| HALLMARK_HEDGEHOG_SIGNALING | 36 | -0.5258074 | -1.7247847 | 0.00996016 | 0.20933092 |
| HALLMARK_PI3K_AKT_MTOR_SIGNALING | 105 | -0.42029977 | -1.583989 | 0.04771372 | 0.40088522 |
| HALLMARK_BILE_ACID_METABOLISM | 112 | -0.39521 | -1.5829207 | 0.023904383 | 0.3216071 |
| HALLMARK_MYOGENESIS | 200 | -0.3872878 | -1.5657897 | 0.031185031 | 0.29902706 |
| HALLMARK_HEME_METABOLISM | 197 | -0.37981457 | -1.5604057 | 0.020920502 | 0.26622048 |
| HALLMARK_G2M_CHECKPOINT | 198 | -0.53517824 | -1.5309132 | 0.12277228 | 0.27279952 |
| HALLMARK_APICAL_JUNCTION | 200 | -0.39452493 | -1.5223526 | 0.07855626 | 0.2562285 |
| HALLMARK_DNA_REPAIR | 149 | -0.44854432 | -1.5076077 | 0.09829868 | 0.24600564 |

Table S2 The top 10 most enriched Hallmark terms in GSEA
